# Supplementary material for: Membrane cholesterol access into a G-protein-coupled receptor
Source: Nat Commun. 2017 Feb 21;8:14505. doi: 10.1038/ncomms14505 (PMC5321766; doi:10.1038/ncomms14505)
Supplement: Supplementary Information — Supplementary Figures, Supplementary Tables, Supplementary Notes, Supplementary Discussion, Supplementary References. [file ncomms14505-s1.pdf]

Supplementary Notes and Discussion

Supplementary note 1 - Both orthosteric and allosteric effect contribute to the cholesterol-mediated modulation of $A_{2A}R$ specific binding

As previously postulated for other GPCRs, cholesterol is thought to modulate ligand binding from the exterior of the protein via specific binding at the surface of the receptor (i.e. direct allosteric effect) or by changing key membrane properties like fluidity or membrane thickness (i.e. indirect allosteric effect) (reviewed in refs. [10] and [11]). In this line, our molecular dynamics simulations corroborate the presence of several interacting sites at the surface of the $A_{2A}R$ (Supplementary Fig. 10), as previously suggested by Lee & Lyman[12]. Likewise, new saturation binding experiments (Fig. 3) show traits that point towards de ability of cholesterol to allosterically modulate ligand binding (e.g. major increase in B_{max} and slight increase in K_d upon cholesterol depletion). On the other hand, competitive binding experiments in the presence of increasing WSC concentrations (1 µM to 3 mM) (Fig. 4) suggest an orthosteric mode of action of cholesterol. In these experiments, WSC is able to displace 20 nM [^{3}H]ZM241385 binding with an equilibrium dissociation constant (Ki) of 233 µM (95% confidence intervals 194 to 279 µM), confirming the ability of cholesterol to compete for binding to the orthosteric site.
Nevertheless, classical binding experiments are probably not able to separate orthosteric from allosteric contribution when both modulations are exerted together. In addition, cholesterol is generally considered to bind the surface of membrane proteins (or at most at shallow sites) and hence deeply buried sites are simply overlooked, as shown by Brannigan et al. for the nicotinic acetylcholine receptor[13]. The same assumption has likely precluded the observation of cholesterol accessing the interior of the protein in some of the previously reported cholesterol-mediated modulation of GPCRs. Overall, while the present study represents, to our knowledge, the first report of a competitive effect of cholesterol at the orthosteric site of a GPCR, future investigations shall aim to unveil the exact contribution of each type of cholesterol modulation (i.e. orthosteric versus allosteric) at the $A_{2A}R$ or other receptors of the family.

Supplementary note 2 - Effect of inhibition of receptor internalization on $A_{2A}R$ specific binding

Cholesterol depletion has been shown to alter the structure of both clathrin-coated pits[1–3] and cholesterol-rich caveolae[4,5] thus inhibiting endocytosis. This inhibitory effect on the formation of endocytic vesicles could interfere with normal $A_{2A}R$ trafficking (i.e. modify receptor concentration in the membrane) thus altering the outcome of specific binding assays. In order to rule out this possibility, we studied $A_{2A}R$ specific binding in the presence of two different cell permeable inhibitors, namely Pitstop 2 and Dynasore. Dynasore is a dynamin GTPase inhibitor that prevents the scission of dynamin-dependent endocytic vesicles (i.e. clathrin- and caveolin-coated vesicles)[6,7]. On the other hand, whilst Pitstop 2 has been commercialized as clathrin-mediated endocytosis inhibitor[8], it has also been shown to inhibit clathrin-independent endocytosis[9]. As we show in Supplementary Fig. 8, neither Dynasore (bars 3 and 4) nor Pitstop 2 (bar 5) was able to significantly modulate $A_{2A}R$ specific binding after 20-40 minutes when compared to the control (bar 1). This clearly demonstrates that the increase in specific binding upon cholesterol depletion via MβCD treatment (Supplementary Fig. 8, bar 2) is not due to an inhibition of receptor internalization.

Supplementary note 3 - Biotinylation experiments

1. **Details about the methodology:** originally, the Substituted-Cysteine Accessibility Method (SCAM) was developed to elucidate water-accessible residues in membrane-spanning proteins like channels[14], transporters[15] or binding-site crevices[16]. In the absence of crystallography data, this method requires a systematic mutation of every protein residue into a cysteine followed by an assessment of ligand binding properties. Throughout a decade, the SCAM method has been employed by Javitch et al. to explore water-accessible residues in one G protein coupled receptor, namely the dopamine D_2 receptor (D_2R)[16–18]. As mentioned in this work, an ideal starting point would be to create a cysteine-free pseudo-wild-type background that is insensitive to the reagents and has normal expression and function. However, intense efforts failed to achieve such construct for the D_2R[19]. It is likely that the lack of cysteine residues in cysteine-free pseudo wild type GPCRs impacts protein expression, folding and thus the formation of a functional receptor.

   In the present study, we adapted the SCAM method to explore the reactivity of cysteine residues under different conditions in the $A_{2A}R$ interior. The basis of the employed methodology relies on two key aspects:

   a. Methanethiosulfonate (MTS) reagents react with sulfhydryl groups of accessible cysteine residues[19]. Thus, only cysteines at the water-accessible surface of the receptor, namely the extracellular side or interior of the membrane-spanning segment, will be reactive to MTS reagents (i.e. cysteine residues at the lipid-accessible surface will not be reactive).
   b. MTS reagents are specific for free sulfhydryl groups, and thus residues engaged in a disulfide bridge are not reactive even if they are accessible to water[19,20].

   In contrast to the work from Javitch et al., in the present study we know the exact location of water-accessible cysteines in the $A_{2A}R$ thanks to the availability of high resolution crystallography data (PDB:3EML). This knowledge is crucial for correct interpretation of biotinylation and binding experiments as outlined below.

2. **Ability of cysteine residues to react with MTSEA-B in the $A_{2A}R$.** In order to assess the ability of the $A_{2A}R$ to react with the biotinylation reagent MTSEA-B, the receptor was scanned for cysteine residues, their location and engagement in disulfide bridges. As shown by high resolution crystallography data (PBD: 3EML) (Supplementary Fig. 14, Supplementary Table 5), 8 out of 14 endogenous cysteines residues are engaged in 4 disulfide bridges at the extracellular side of the receptor, and thus these 8 residues are not susceptible to be biotinylated (see point **1b** above). The 6 remaining cysteines are located in the transmembrane portion of the protein, and thus they can only face either the membrane (2 cysteines) or the interior of the receptor (4 cysteines). Since lipid-accessible cysteine residues are not reactive (see point **1a** above), there are only 4 cysteines in the receptor interior which can potentially react with the biotinylation reagent (C3.30, C4.49, C5.46, C6.56) (Supplementary Fig. 14, Supplementary Table 5). A structural inspection of those cysteines shows that C4.49 is buried between TM3 and TM4 (i.e. not water-accessible) yielding only 3 reactive cysteines in the receptor interior that are susceptible to be biotinylated, namely C3.30, C5.46, and C6.56. As shown in Supplementary Fig. 15, chemical modification of these cysteine residues by MTSEA-B would clearly overlap with the orthosteric binding site of the $A_{2A}R$.

In summary, a detailed structural characterization of the $A_{2A}R$ suggests that all cysteines outside the receptor are non-reactive as they are engaged in disulfide bridges and that only cysteines inside the receptor are reactive towards the biotinylation reagent MTSEA-B. Hence, regardless of which cysteines become biotinylated in the receptor interior, cholesterol needs to enter the receptor interior to exert its action. It is worth noting that based on the experimental set-up used in this paper, we cannot pinpoint the exact cysteine residue reacting with the biotinylation reagent. However, provided that MTSEA-B should access the receptor from the extracellular side, we can speculate that the first cysteine residue (i.e. C3.30) found on the MTSEA-B entrance pathway into receptor should mostly react with the biotinylation reagent.

Supplementary note 4 - Exploring exit pathways for cholesterol from $A_{2A}R$ ligand binding site

Methyl-β-cyclodextrin (MβCD), the cholesterol-depleting agent we use in our experiments, is not likely to remove cholesterol from the interior of the $A_{2A}R$ pocket. Thus, it is reasonable to think that cholesterol first has to leave spontaneously the interior of the $A_{2A}R$ towards the membrane bulk before being removed by MβCD. In this context, we explored the energetic cost of extracting cholesterol from the $A_{2A}R$ binding crevice into the aqueous phase. As we discuss in the main text, the extraction of cholesterol to the water phase is very costly, namely 120 kJ·mol^{-1} (~50 K_BT). Due to its hydrophobic moiety, cholesterol is highly reluctant to become exposed to the water phase but rather establishes a strong interaction with the extracellular loop 2 of the protein (see Supplementary Fig. 16, region labeled as C). A visual inspection of our simulations shows that shortly after pulling forces are applied upwards (i.e. ξ within 1-2 nm), cholesterol changes its initial orientation with respect to the membrane plane from a rather fluctuating parallel orientation to a more stable perpendicular position. Interactions between cholesterol hydroxyl group and water molecules present at the interior of the protein somehow shield cholesterol hydrophobic ring and play an important role in the position and stability of cholesterol inside and the pocket.

As highlighted in Supplementary Fig. 16, the energy landscape of cholesterol exiting the $A_{2A}R$ towards the water phase can be divided in three main regions by labels **A, B** and **C**. While cholesterol exiting from $A_{2A}R$ through the water phase is unlikely, our PMF calculations show a thermally accessible region which might be a key for a direct exit pathway into the membrane bulk. This region is located between ~20 kJ·mol^{-1} (~8 K_BT) (label **A** in Supplementary Fig. 16) and ~37 kJ·mol^{-1} (~15 K_BT) (label **B** in Supplementary Fig. 16). A visual inspection of the simulations shows that the lowest energy barrier of this region (i.e. label **A**) corresponds to cholesterol rigid ring progressing through the space limited by TM2 and TM7. Once cholesterol overcomes this barrier, the molecule is ready to head towards TM1-TM2 and/or TM1-TM7 exit to the membrane bulk. But in order to proceed to any of these exit gates, cholesterol also tail needs to cross the TM2 -TM7 corridor which involves an extra energy cost (i.e. label **B**). Label **C** mainly corresponds to the cholesterol molecule in the water phase, mostly interacting with the extracellular loop 2 of the $A_{2A}R$.

In addition, as shown in label **A** of Supplementary Fig. 16, the position of cholesterol oxygen along the z axis in this region is very similar to that of membrane cholesterol bulk (reference density profile, blue line) which would eventually allow a comfortable insertion of cholesterol into the membrane upon exiting the receptor via TM1-TM2 and/or TM1-TM7. Interestingly, previous work[21] on the β_2-adrenergic receptor using random acceleration molecular dynamics have highlighted that TM1-TM2 and TM1-TM7 are potential ligand entry/exit pathways. It is important to note that during our simulations the head of two phospholipids from the upper leaflet of the membrane (i.e. extracellular) significantly occupy both TM1-TM2 and TM1-TM7 grooves, as previously described in other computational and experimental studies.

This suggests that exit via TM1-TM2 and TM1-TM7 grooves may be additionally modulated by the diffusion of these phospholipids, an event way beyond our simulated time scale

Supplementary Tables

Supplementary Table 1 | Effect of cholesterol presence on different GPCRs

Receptor	Ligand binding	Functional outcome
α_{1A}-adrenergic	D[22]	D[22]
β_2-adrenergic	E[23]	E[24]
Adenosine A_{2A}	**D***<br>E[25,26], or N[27]	D[28]<br>E[29], or N[29]
Cannabinoid CB_1	D[30,31]	D[30,31]
Cannabinoid CB_2	N[32]	N[32]
Chemokine CCR_5	E[33,34]	E[33,34]
Chemokine CXR_4	E[34,35]	E[34,35]
Cholecystokinin CCK_1	E[36,37]	E[36]
Cholecystokinin CCK_2	N[38]	N[38]
Dopamine D_1	D[39]	D[39]
Galanin	E[40]	E[40]
Metabotropic glutamate	E[41]	E[42]
Muscarinic M_2	D[43,44]	D[44]<br>E[44], or N[44,45]
Neurotensin NTS_1	N[46]	Unknown
Neurokinin NK_1	E[47]	E[47]
μ-opioid	E[48,49], or N[49]<br>D[50]	E[48,49]
δ-opioid	E[51]<br>N[48]	D[51], or E[51]<br>N[48]
κ-opioid	D[52]	D[52]
Oxytocin	E[36,53–55]	E[36]
Rhodopsin	Unknown	D[56–58]
Serotonin $5HT_{1A}$	E[59–61]<br>D[62–64]	E[59–61]<br>D[62–64]
Serotonin $5HT_{7A}$	E[65]	E[65]

*** Results reported at the present manuscript**
Abbreviations: Enhanced (E), Diminished (D), and No effect (N)

Supplementary Table 2 | List of simulations performed

Simulation set	Simulation time (μs)	Number of replicates	Purpose
1	1	4	Cholesterol-mediated modulation of $A_{2A}R$ (Initial set of simulations)
2	0.1	40	Cholesterol invasion of $A_{2A}R$ (original membrane composition as in Supplementary Table S2)
3	0.1	40	Influence of membrane environment on cholesterol invasion of $A_{2A}R$
4	0.1	40	Validation of ICL3 inclusion
5	10	3	Behavior of cholesterol inside $A_{2A}R$
6	8 (200-600 ns/window)	35 windows	Cholesterol exit of $A_{2A}R$ (PMF)

Supplementary Table 3 | Composition of simulation systems

Component	Number of molecules
DPPC	48
DSPC	18
DOPC	36
SDPC	44
SM	79
CHOL	112
Total	337
$A_{2A}R$	1
Water	22908
Na+	65
Cl-	75

Abbreviations: 1,2-dipalmitoyl-sn-glycero-3-phosphocholine (DPPC), 1,2-distearoyl-sn-glycero-3-phosphocholine (DSPC), 1,2-dioleyl-sn-glycero-3-phosphocholine (DOPC), 1-stearoyl-2-docosahexaenoyl-sn-glycero-3-phosphocholine (SDPC) and sphingomyelin (SM), cholesterol (CHOL), and water (W), respectively.

Supplementary Table 4 | Distance to the protein interior from short replicates

Group of replicates	Complex membrane	ICL3 included	POPC bilayer
1-10	32.78 ± 0.45	33.48 ± 0.12	38.12 ± 0.82
11-20	28.08 ± 0.48	29.43 ± 0.16	35.99 ± 0.60
21-30	20.28 ± 0.15	21.18 ± 0.10	24.76 ± 0.40
31-40	20.98 ± 0.36	19.19 ± 0.14	22.43 ± 0.36

As in Fig. 6, and Supplementary Figs. 12, and 13, values are reported as the mean distance from the center of mass of cholesterol to protein residue E1.39 (Å) ± the standard error of the mean (s.e.m.). The last 50 ns of each trajectory were used in this analysis. The initial distance for replicates 1-10, 11-20, 21-30 and 31-40 is 32.05 Å, 31.60 Å, 21.23 Å and 19.80 Å, respectively (depicted as red horizontal lines in Supplementary Fig. 6, and Supplementary Figs. 12, and 13).

Supplementary Table 5 | Location and water-accessibility $A_{2A}R$ cysteine residues*

Residue ID	Location	Water-accessible
**C3.30**	TM3	**Yes**
**C5.46**	TM5	**Yes**
**C6.56**	TM6	**Yes**
C1.54	Lower TM1	No
C4.49	Lower TM4	No
C6.47	TM6	No
C71	EC**	No
C74	EC**	No
C76	EC**	No
C146	EC**	No
C159	EC**	No
C166	EC**	No
C259	EC**	No
C262	EC**	No

*** Water-accessible cysteines highlighted in bold**
**** EC: extracellular loops (locked in disulfide bridge)**

Supplementary Figures

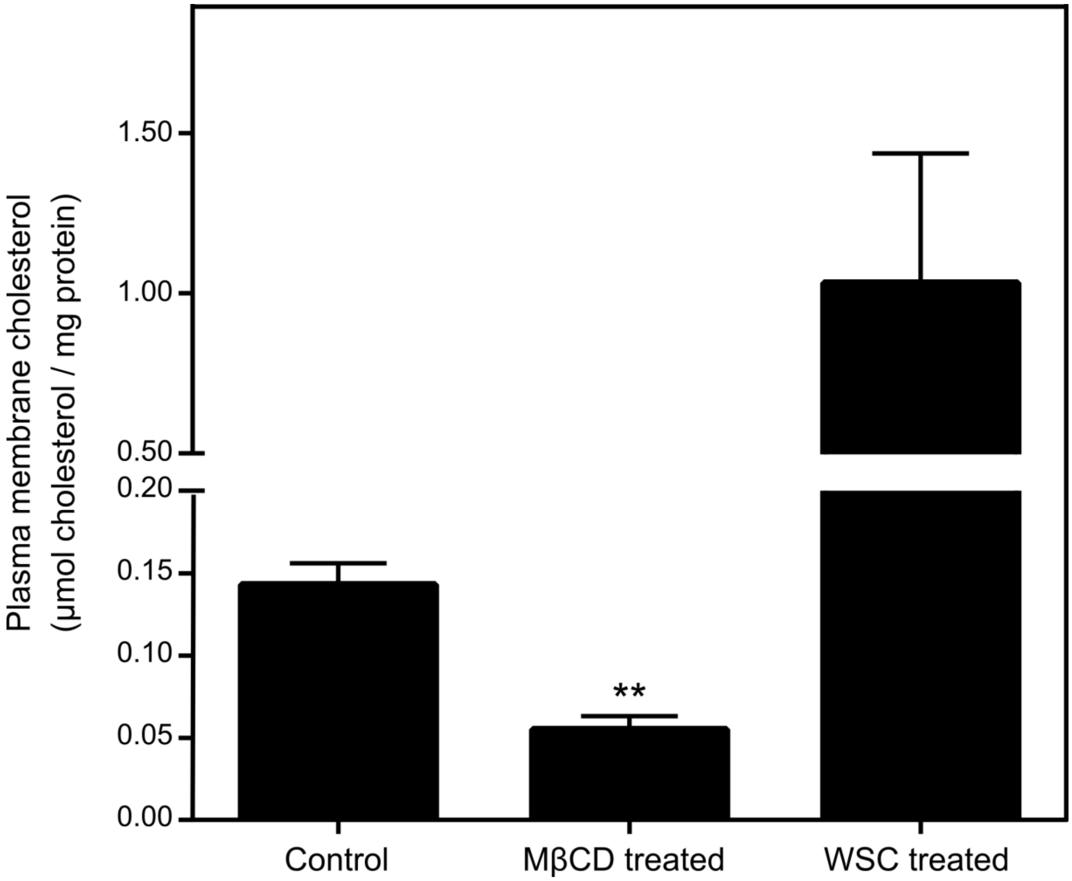

Supplementary Figure 1 | Cholesterol quantification in C6 plasma membranes. Cholesterol (Ch) concentration was measured by targeted lipidomics in plasma membranes isolated from control (n=3), 5 mM MβCD (40 minutes, n=2) and 1 mM WSC (50 minutes, n=2) treated cells as described in the Methods. Data are mean ± s.e.m. values from indicated n samples. ** p<0.01 significantly different from control value according to a Student's t-test.

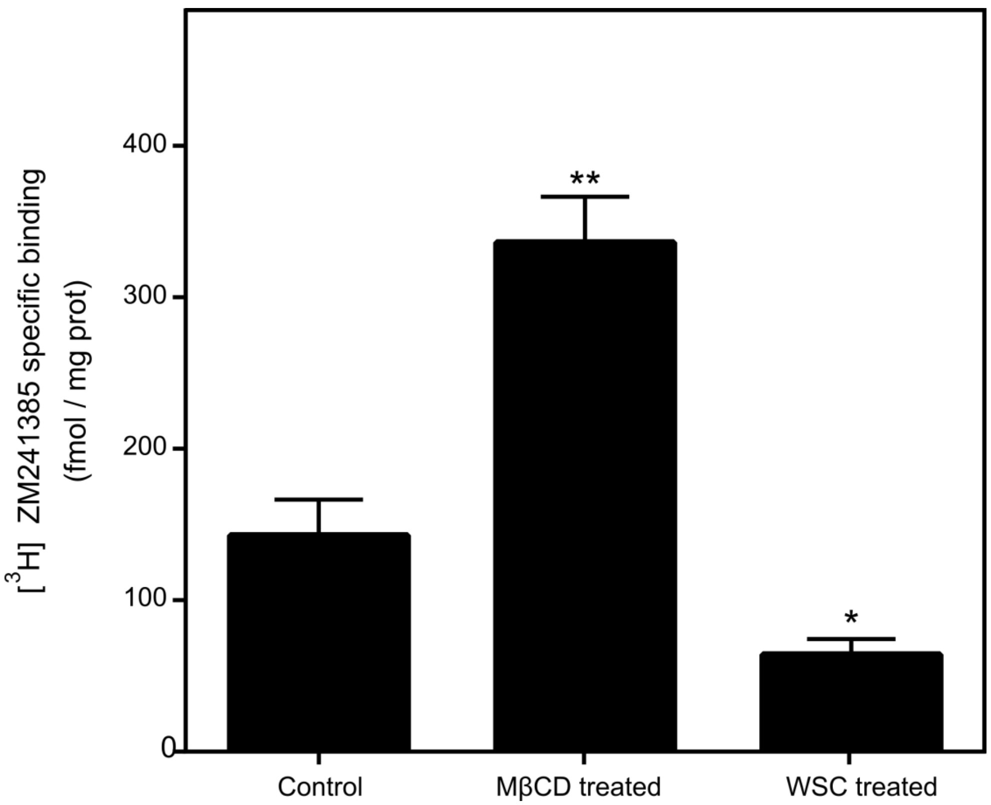

Supplementary Figure 2 | Effect of MβCD and WSC on specific A_{2A}R binding in C6 plasma membranes. Plasma membranes were isolated from control (n=3), 5 mM MβCD (40 minutes, n=3) and 1 mM WSC (50 minutes, n=2) treated cells. Specific [^{3}H]ZM241385 binding (i.e. 40 nM) to A_{2A}R was measured as detailed in the Methods. These results are mean ± s.e.m. values obtained from the indicated number samples (n) analyzed in triplicate. * $p<0.05$ and ** $p<0.01$ significantly different from control value according to a Student's t-test.

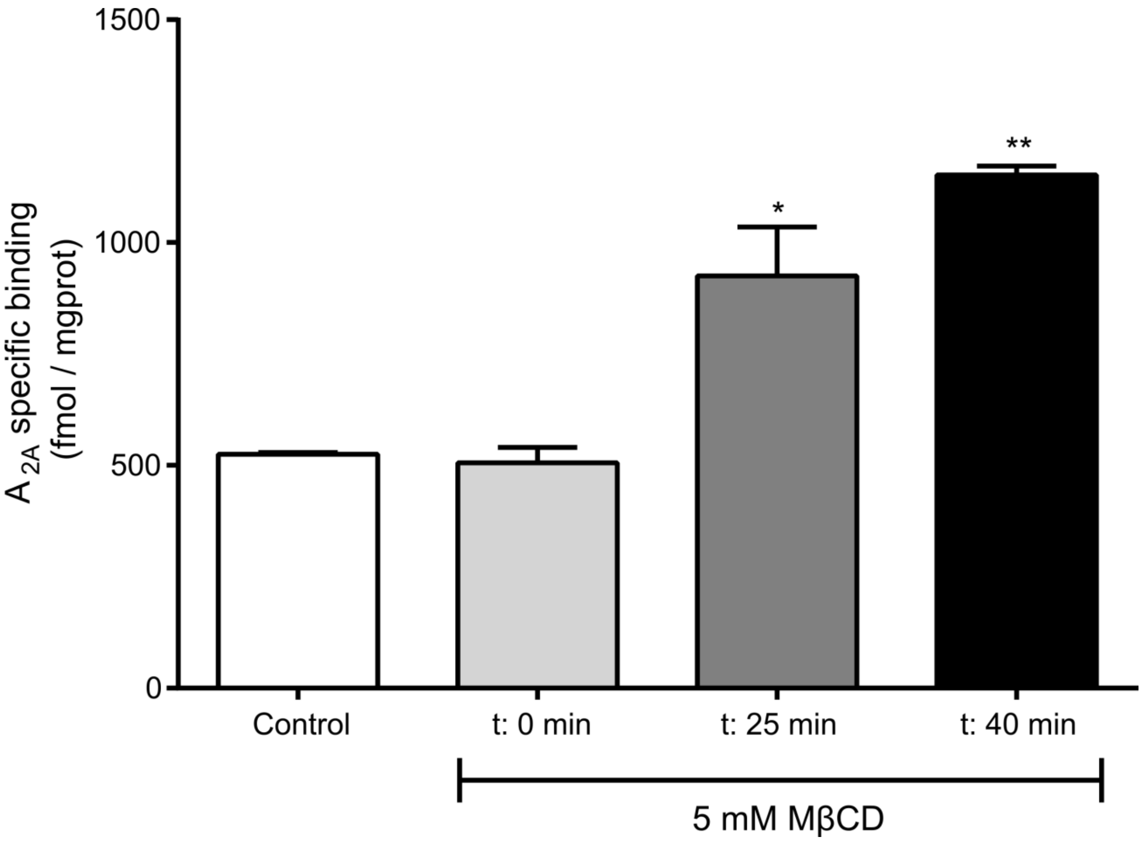

Supplementary Figure 3 | Effect of MβCD on specific A_{2A}R binding in intact C6 glioma cells. Cells washing after MβCD treatment was satisfactory as no differences were detected between values from control and fast (seconds) MβCD adding and washing (t:0) conditions. Increased binding to A_{2A}R was detected at 25 minutes (t:25) and 40 minutes (t:40) of treatment. These results are mean ± s.e.m. values obtained from n=3 (columns 1, 2 and 4) and n=4 (column 3) separate experiments carried out in triplicate. * $p<0.05$ and ** $p<0.01$ significantly different from control value according to a Student's t-test.

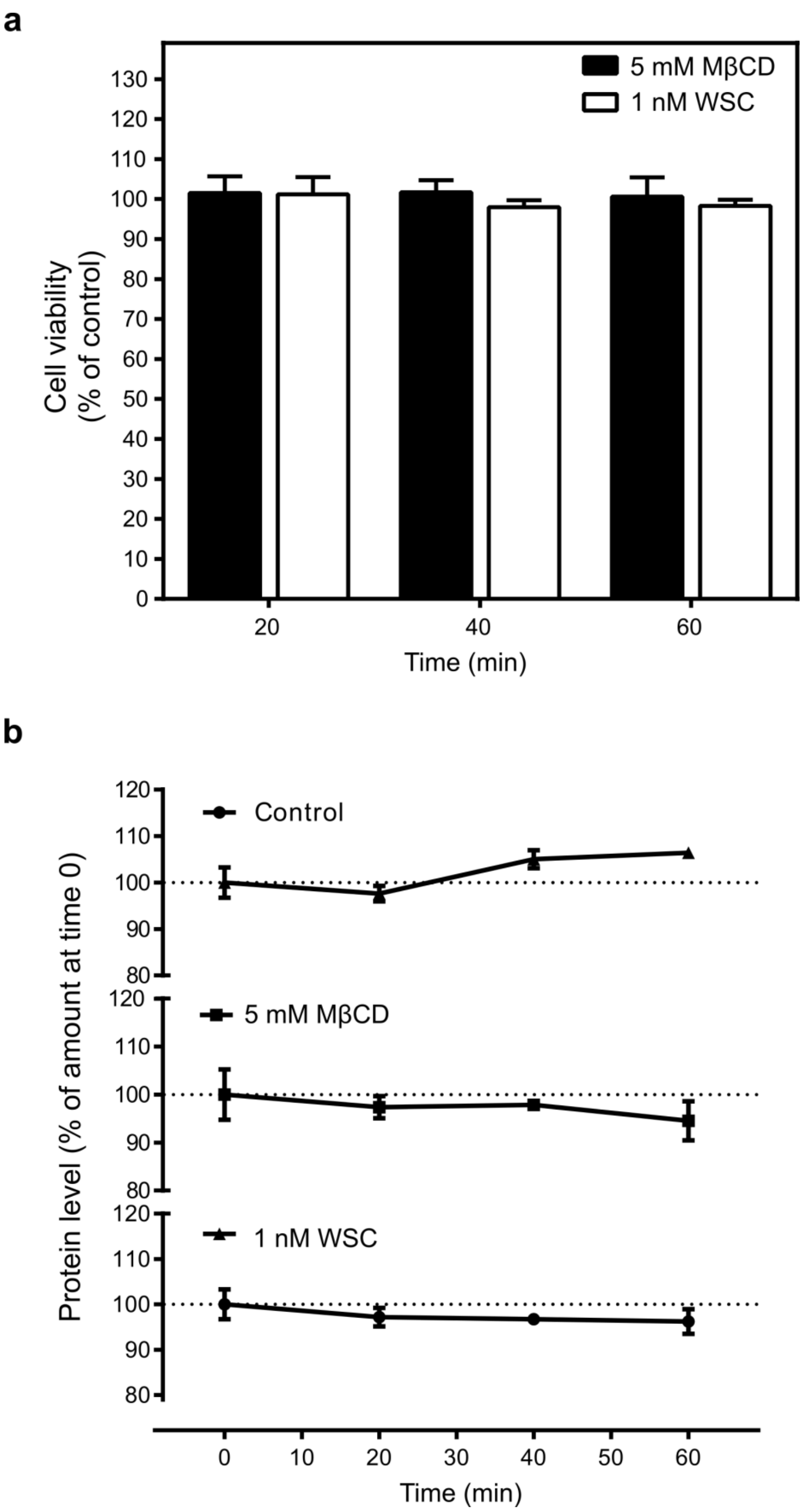

Supplementary Figure 4 | Effect of MβCD or WSC treatment on C6 cells viability and protein level. Cell viability (**a**) and total protein amount (**b**) were measured in C6 intact cells at indicated time of treatment and compared to control cells values. These results are mean ± s.e.m. values obtained from 4 independent assays carried out in sextuplicate **(a)** or duplicate **(b)**.

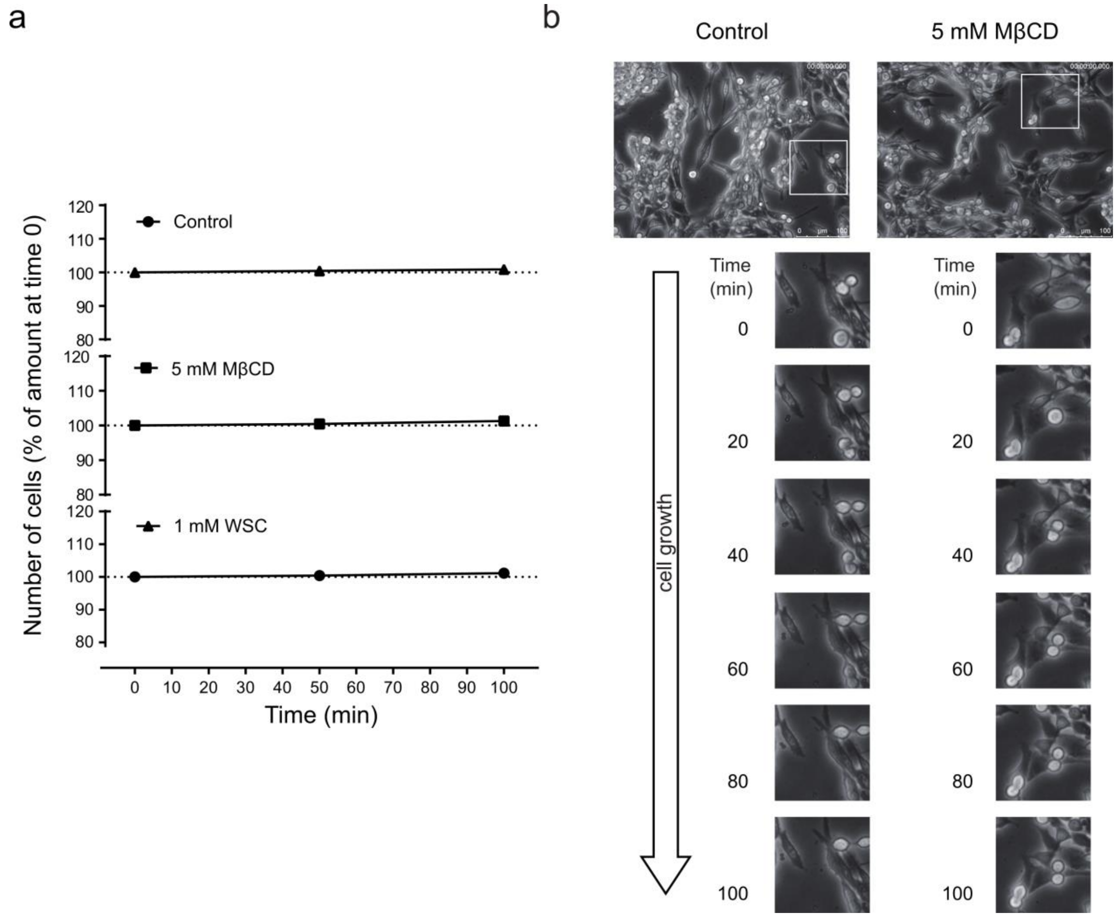

Supplementary Figure 5 | Effect of MβCD or WSC treatment on C6 cells growth. (a) Cells were counted from phase contrast images recorded (1 frame/minute) for 100 minutes at the indicated interval and relative to the number of cells at the beginning of treatment. These results are mean ± s.e.m. values obtained from 2 independent assays performed at different cell passage. **(b)** Cell morphology does not significantly change during MβCD treatment (Scale = 100 μm).

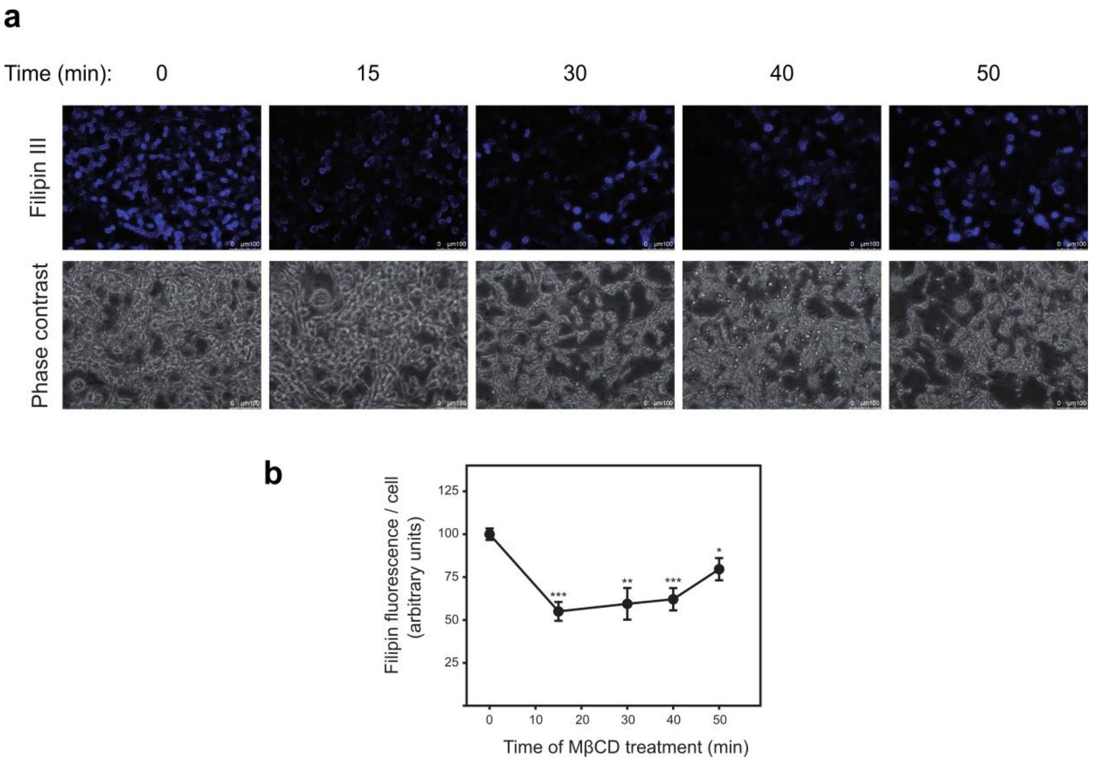

Supplementary Figure 6 | Cholesterol depletion from membranes monitored by filipin III staining. Phase contrast and filipin III fluorescence images of intact C6 glioma cells (100 μm scale bars plotted in the bottom right corner of each image) **(a)** and quantitation of filipin III fluorescence intensity **(b)** after treatment with 5 mM MβCD during the indicated time. Since filipin III specifically binds cholesterol, quantitation of filipin III fluorescence intensity monitors here membrane cholesterol depletion. These results are mean ± s.e.m. values obtained from n=5 separate experiments carried out in different cell culture passages. * $p<0.05$, ** $p<0.01$ and *** $p<0.001$ significantly different from control value (t:0) according to a Student's t-test.

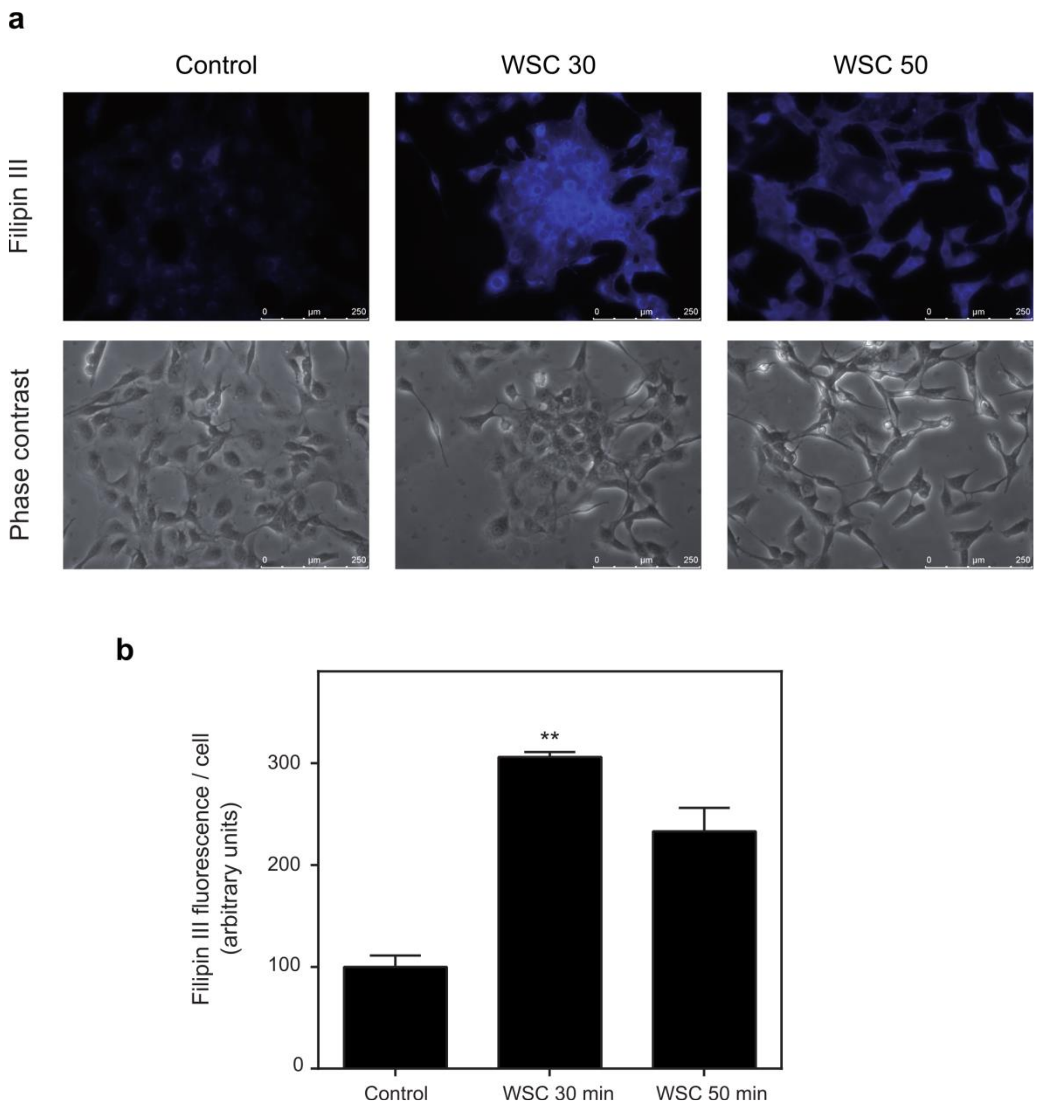

Supplementary Figure 7 | Cholesterol replenishment to intact cell membranes monitored by filipin III staining. Phase contrast and filipin III fluorescence images (Scale = 250 µm) of intact C6 glioma cells **(a)**, and quantitation of filipin III fluorescence intensity **(b)** after treatment with 1 mM water soluble cholesterol (WSC) during the indicated time. Since filipin III specifically binds cholesterol, an adequate incorporation of cholesterol into the membrane is followed by an increase in filipin III fluorescence intensity. These results are mean ± s.e.m. values obtained from 3 separate experiments carried out in different cell culture passages. ** $p<0.01$ significantly different from control value according to a Student's t-test.

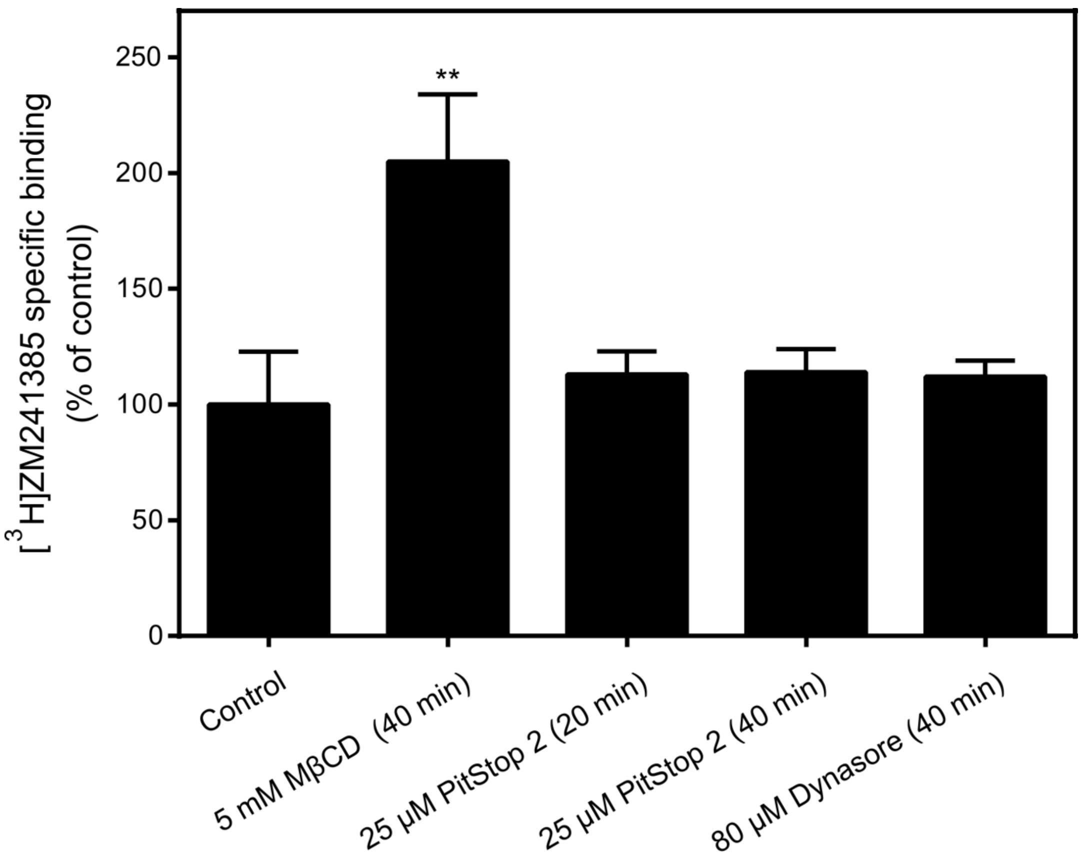

Supplementary Figure 8 | Effect of MβCD or different endocytosis inhibitors on specific $A_{2A}R$ binding in intact C6 glioma cells. Cells were treated for the indicated time (minutes) with MβCD or endocytosis inhibitors Pitstop 2 or Dynasore. Specific binding of 20 nM [^{3}H]ZM241385 to A_{2A}Rs was determined as described in the Methods. These results are mean ± s.e.m. values obtained from n=4 (columns 2, 3 and 5), n=5 (column 4) and n=6 (column 1) separate experiments carried out in duplicate. ** $p<0.01$ significantly different from control value according to a one-way ANOVA test.

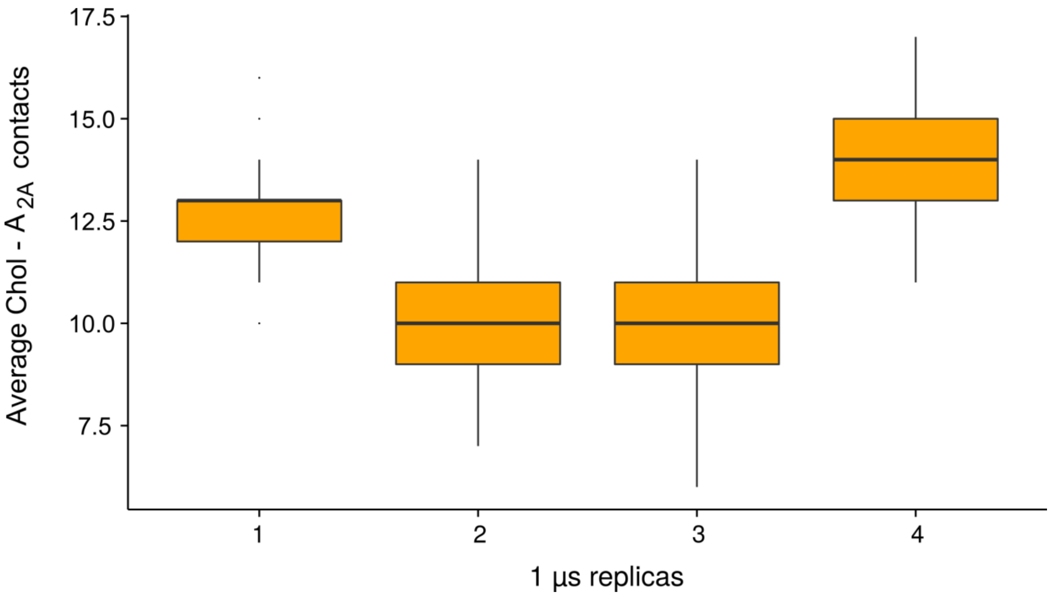

Supplementary Figure 9 | Average number of cholesterol- $A_{2A}R$ contacts. Average number of cholesterol molecules within a 2.9 Å distance of the $A_{2A}R$ in 4 separate replicates of 1 µs (x axis).

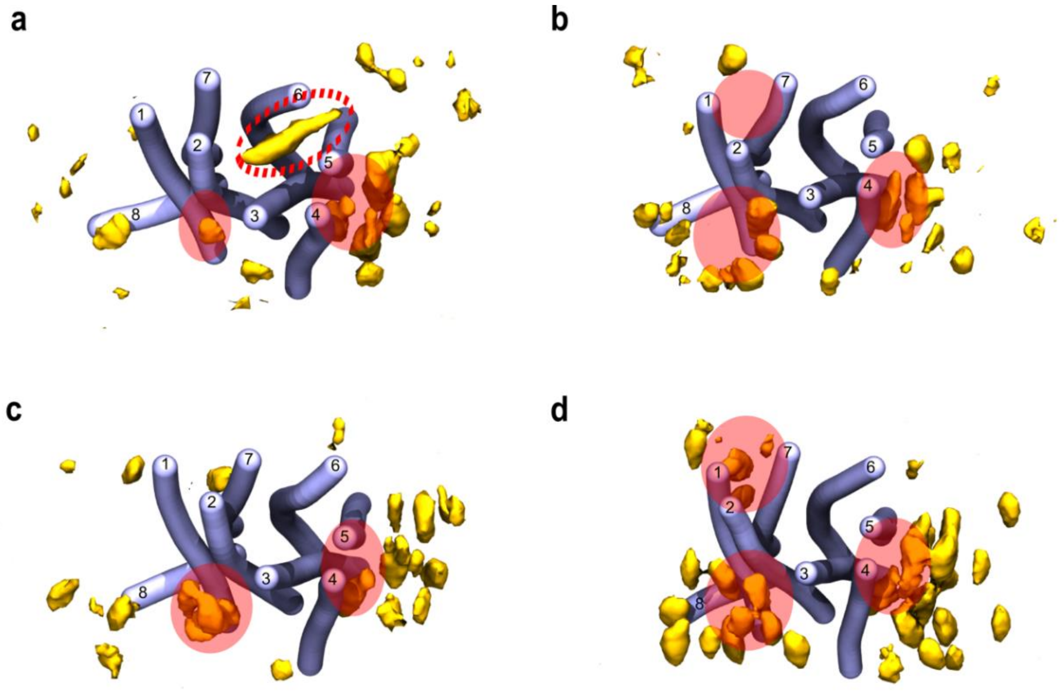

Supplementary Figure 10 | Cholesterol density around the $A_{2A}R$. Volumetric map of the average cholesterol density (yellow) around the $A_{2A}R$ (blue) for each 1 µs simulation (**a**, **b**, **c**, and **d** display replicates 1, 2, 3 and 4, respectively). Key surface contact areas (i.e. most populated) are highlighted in red-filled circles. Cholesterol inside the receptor is circled by a red-dashed line. The BENDIX[66] plugin for VMD was used to depict protein helices. Protein loops were omitted for clarity.

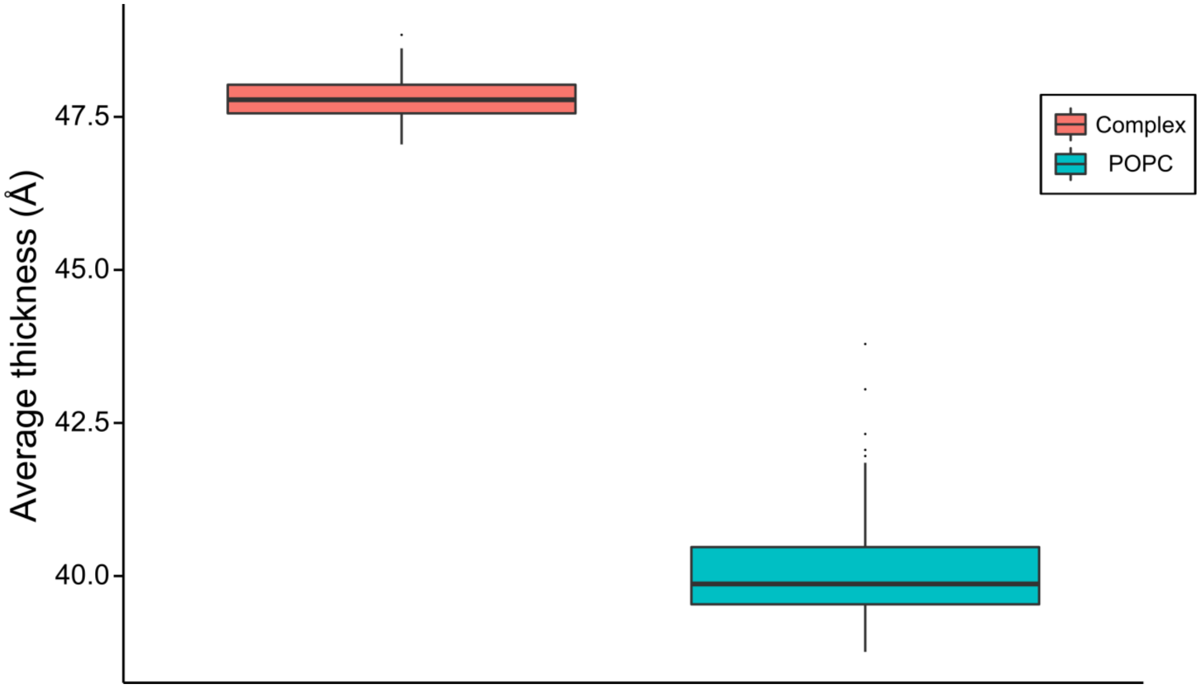

Supplementary Figure 11 | Membrane thickness of complex and POPC membranes. Average membrane thickness (Å) (i.e. distance between phosphorous atoms) was computed using the MEMBPLUGIN[67] of VMD. All trajectories analyzed in this manuscript (see Supplementary Table 2) were used to obtain average values.

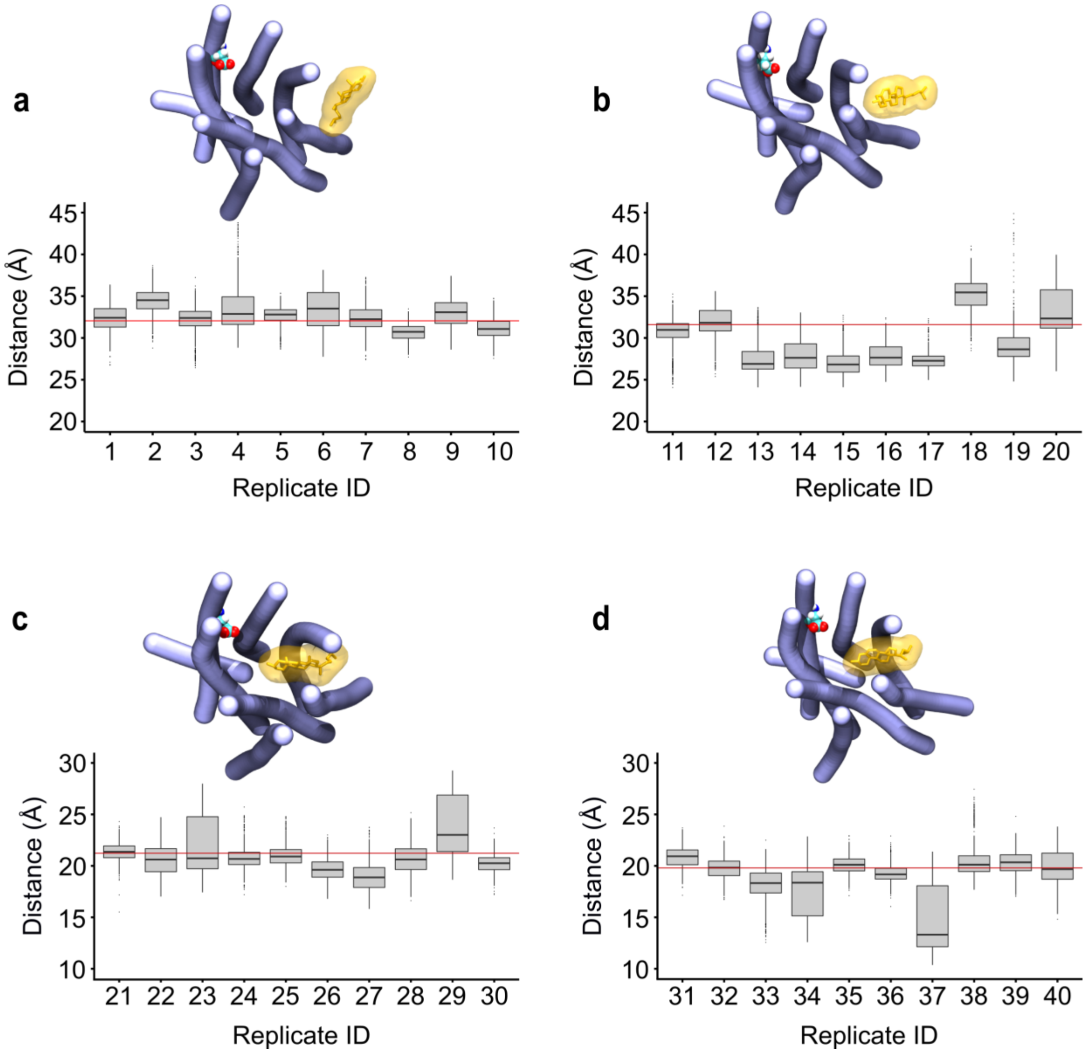

Supplementary Figure 12 | Short simulation replicas of cholesterol entrance (ICL3 included). Short simulations of the $A_{2A}R$ (ICL3 included, as described in the methods and SI methods) and target cholesterol embedded into the original bilayer (see Supplementary Tables 2 and 3). As in Figs. 6, boxplots display the distance between the center of mass of cholesterol and residue E1.39 for a set of 40 replicate simulations of 100 ns. 4 different starting positions (**a, b, c and d**) re-spawned from the original cholesterol entrance trajectory were used to run each 10 replicates (i.e. 1-10, 11-20, 21-30, 31-40). The red horizontal line of each graph corresponds to the distance at the beginning of the simulation as measured from the snapshot used to re-spawn each set of 10 trajectories. Average distance for each set of replicates is reported in Supplementary Table 4. Inset figures show the initial structure of the $A_{2A}R$ (in blue) and cholesterol residue (in orange) used to start each set of simulations. E1.39 residue displayed as van der Waals spheres. The BENDIX[66] plugin for VMD was used to depict protein helices. Protein loops were omitted for clarity.

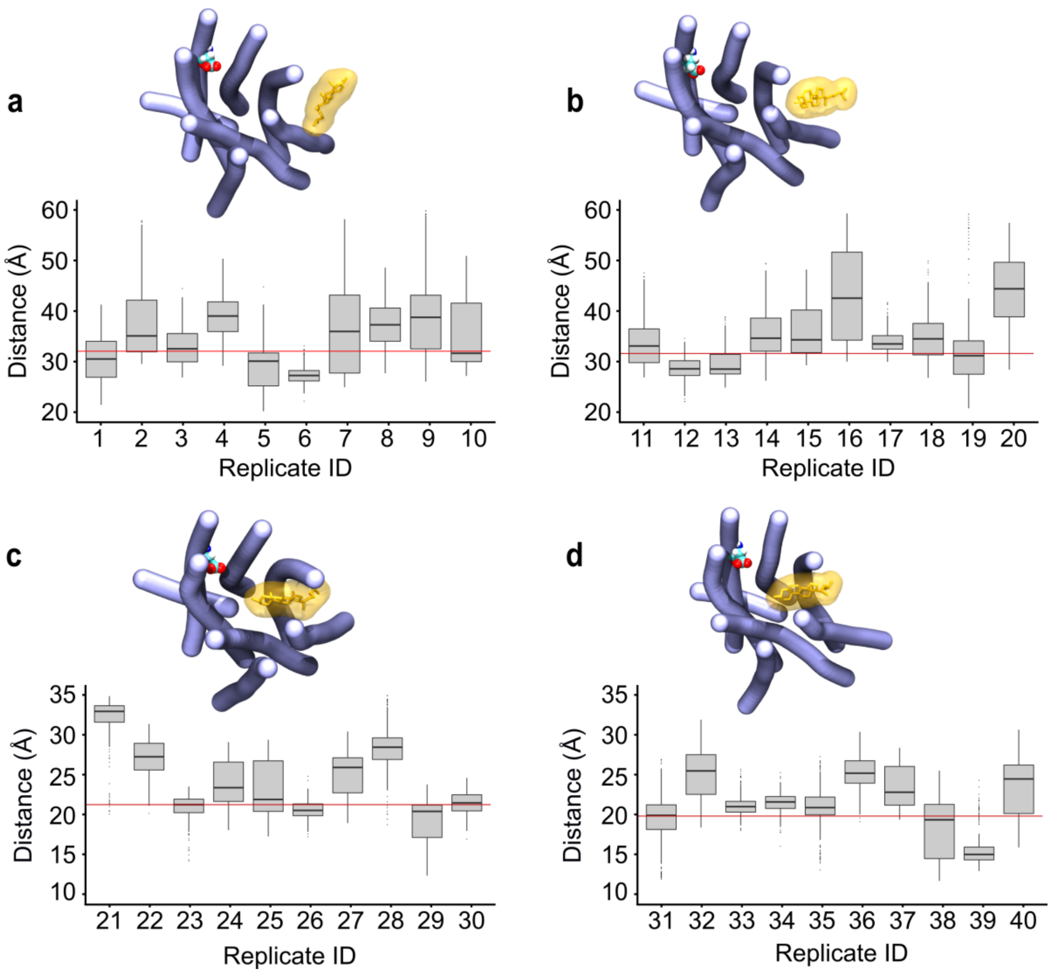

Supplementary Figure 13 | Short simulation replicas of cholesterol entrance (POPC bilayer). Short simulations of the $A_{2A}R$ and target cholesterol embedded into a POPC bilayer. As in Fig 6, each boxplot display the distance between the center of mass of cholesterol and residue E1.39 for a set of 40 replicate simulations of 100 ns. 4 different starting positions (**a, b, c** and **d**) re-spawned from the original cholesterol entrance trajectory were used to run each 10 replicates (i.e. 1-10, 11-20, 21-30, 31-40). The red horizontal line of each graph corresponds to the distance at the beginning of the simulation as measured from the snapshot used to re-spawn each set of 10 trajectories. Average distance for each set of replicates is reported in Supplementary Table 4. Inset figures show the initial structure of the $A_{2A}R$ (in blue) and cholesterol residue (in orange) used to start each set of simulations. E1.39 residue displayed as van der Waals spheres. The BENDIX[66] plugin for VMD was used to depict protein helices. Protein loops were omitted for clarity.

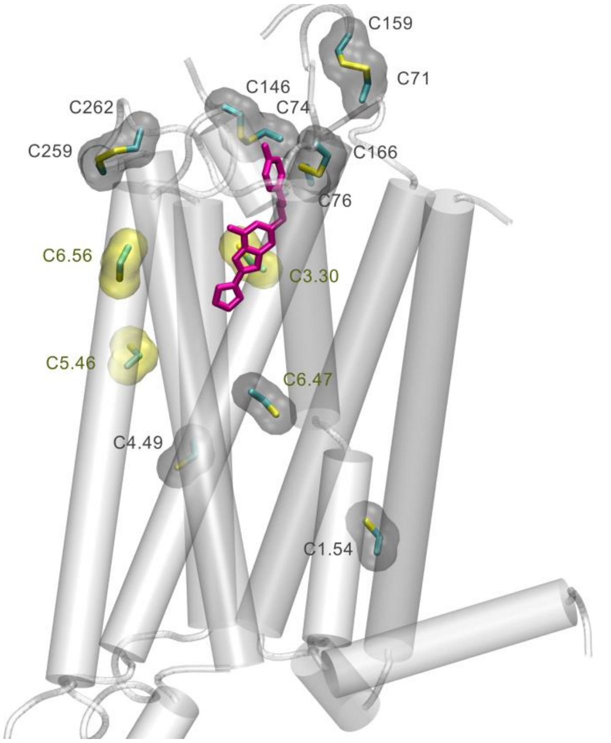

Supplementary Figure 14 | Location of cysteine residues in the $A_{2A}R$ and accessibility to chemical modification via MTSEA-B. Cysteine residues (liquorice representation) susceptible to biotinylation in the $A_{2A}R$ (grey cartoons) are shown in yellow transparent surface whereas non-reactive residues are shown in grey transparent surface. The antagonist ZM241385 is shown in red liquorice. The PDB:3EML structure was used to generate this figure.

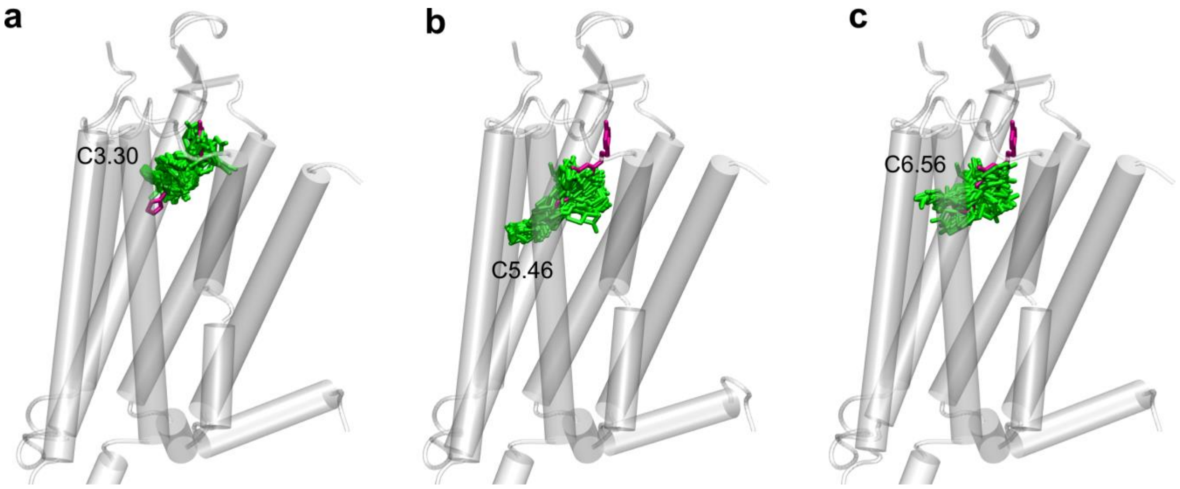

Supplementary Figure 15 | In silico model of MTSEA-B chemical modification of $A_{2A}R$ cysteine residues. (a-c) Chemical modification of the three cysteines (C3.30, C5.46, C6.56) in the binding pocket shows that the covalently bound biotin (green surface) competes with other orthosteric ligands such as ZMA (red stick representation) in the binding pocket.

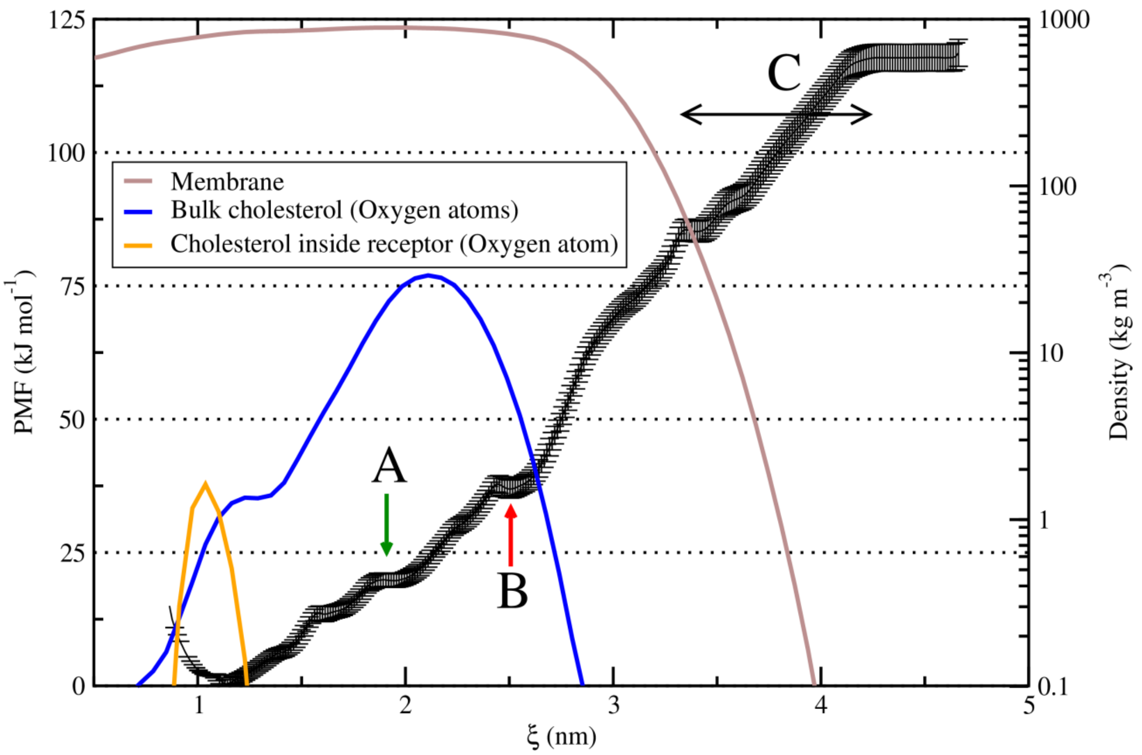

Supplementary Figure 16 | Potential of mean force for the extraction of cholesterol. Cholesterol was extracted from the ligand binding site of the $A_{2A}R$ receptor to the extracellular water phase using umbrella sampling simulations**.** While the black curve is the average free energy difference, standard deviation values obtained from bootstrapping are shown as black error bars. The reaction coordinate (ξ) corresponds to the distance along the z axis between the centre of mass of the $A_{2A}R$ backbone and the oxygen atom of cholesterol. Maroon, blue and orange lines show reference mass density profiles (kg m^{-3}, right y axis) of the whole membrane (all lipid molecules), membrane cholesterol bulk (cholesterol oxygen atoms), and cholesterol at the binding site (cholesterol oxygen atom, data taken from one unbiased simulation), respectively.

Supplementary References

1. Parton, R. G. & del Pozo, M. a. Caveolae as plasma membrane sensors, protectors and organizers. *Nat. Rev. Mol. Cell Biol.* **14,** 98–112 (2013).
2. Lingwood, D. & Simons, K. Lipid rafts as a membrane-organizing principle. *Science* **327,** 46–50 (2010).
3. Guo, S. *et al.* Selectivity of commonly used inhibitors of clathrin-mediated and caveolae-dependent endocytosis of G protein-coupled receptors. *Biochim. Biophys. Acta - Biomembr.* **1848,** 2101–2110 (2015).
4. Subtil, A. *et al.* Acute cholesterol depletion inhibits clathrin-coated pit budding. *Proc. Natl. Acad. Sci. U. S. A.* **96,** 6775–80 (1999).
5. Rodal, S. K. *et al.* Extraction of Cholesterol with Methyl-β-Cyclodextrin Perturbs Formation of Clathrin-coated Endocytic Vesicles. *Mol. Biol. Cell* **10,** 961–974 (1999).
6. Merrifield, C. J., Feldman, M. E., Wan, L. & Almers, W. Imaging actin and dynamin recruitment during invagination of single clathrin-coated pits. *Nat Cell Biol* **4,** 691–698 (2002).
7. Pelkmans, L. & Helenius, A. Endocytosis via caveolae. *Traffic* **3,** 311–320 (2002).
8. Von Kleist, L. *et al.* Role of the clathrin terminal domain in regulating coated pit dynamics revealed by small molecule inhibition. *Cell* **146,** 471–484 (2011).
9. Dutta, D. & Donaldson, J. G. Search for inhibitors of endocytosis: Intended specificity and unintended consequences. *Cell. Logist.* **2,** 203–208 (2012).
10. Paila, Y. D. & Chattopadhyay, A. The function of G-protein coupled receptors and membrane cholesterol: specific or general interaction? *Glycoconj. J.* **26,** 711–20 (2009).
11. Song, Y., Kenworthy, A. K. & Sanders, C. R. Cholesterol as a co-solvent and a ligand for membrane proteins. *Protein Sci.* **23,** 1–22 (2014).
12. Lee, J. Y. J. & Lyman, E. Predictions for Cholesterol Interaction Sites on the A2A Adenosine Receptor. *J. Am. Chem. Soc.* **134,** 16512–16515 (2012).
13. Brannigan, G., Hénin, J., Law, R., Eckenhoff, R. & Klein, M. L. Embedded cholesterol in the nicotinic acetylcholine receptor. *Proc. Natl. Acad. Sci. U. S. A.* **105,** 14418–23 (2008).
14. Akabas, M. H., Stauffer, D. a, Xu, M. & Karlin, a. Acetylcholine receptor channel structure probed in cysteine-substitution mutants. *Science* **258,** 307–310 (1992).
15. Karlin, A. & Akabas, M. Substituted-Cysteine Accessibility Method. *Methods Enzymol.* **293,** 123–145 (1998).
16. Javitch, J. A., Fu, D., Chen, J. & Karlin, A. Mapping the binding-site crevice of the dopamine D2 receptor by the substituted-cysteine accessibility method. *Neuron* **14,** 825–831 (1995).
17. Javitch, J. a., Fu, D. & Chen, J. Residues in the fifth membrane-spanning segment of the dopamine D2 receptor exposed in the binding-site crevice. *Biochemistry* **34,** 16433–16439 (1995).
18. Fu, D., Ballesteros, J. a, Chen, J. & Javitch, J. a. Residues in the Seventh Membrane-Spanning Segment of the Dopamine D2 Receptor Accessible in the Binding-Site Crevice. *Biochemistry* **35,** 11278–11285 (1996).
19. Liapakis, G., Simpson, M. M. & Javitch, J. a. The substituted-cysteine accessibility method (SCAM) to elucidate membrane protein structure. *Curr. Protoc. Neurosci.* **Chapter 4,** Unit 4.15 (2001).
20. Javitch, J. a, Li, X., Kaback, J. & Karlin, a. A cysteine residue in the third membrane-spanning segment of the human D2 dopamine receptor is exposed in the binding-site crevice. *Proc. Natl. Acad. Sci. U. S. A.* **91,** 10355–10359 (1994).
21. Wang, T. & Duan, Y. Ligand entry and exit pathways in the beta2-adrenergic receptor. *J. Mol. Biol.* **392,** 1102–15 (2009).
22. Lei, B., Morris, D. P., Smith, M. P. & Schwinn, D. A. Lipid rafts constrain basal alpha-1A-adrenergic receptor signaling by maintaining receptor in an inactive conformation. *Cell. Signal.* **21,** 1532–1539 (2009).
23. Kirilovsky, J. & Schramm, M. Delipidation of a beta-adrenergic receptor preparation and reconstitution by specific lipids. *J. Biol. Chem.* **258,** 6841–6849 (1983).

24. Ben-Arie, N., Gileadi, C. & Schramm, M. Interaction of the beta-adrenergic receptor with Gs following delipidation. Specific lipid requirements for Gs activation and GTPase function. *Eur. J. Biochem.* **176,** 649–654 (1988).
25. O'Malley, M. A., Helgeson, M. E., Wagner, N. J. & Robinson, A. S. The morphology and composition of cholesterol-rich micellar nanostructures determine transmembrane protein (GPCR) activity. *Biophys. J.* **100,** L11-3 (2011).
26. Naranjo, A. N., McNeely, P. M., Katsaras, J. & Robinson, A. S. Impact of purification conditions and history on A2A adenosine receptor activity: The role of CHAPS and lipids. *Protein Expr. Purif.* **124,** 62–67 (2016).
27. Bocquet, N. *et al.* Real-time monitoring of binding events on a thermostabilized human A2A receptor embedded in a lipid bilayer by surface plasmon resonance. *Biochim. Biophys. Acta - Biomembr.* (2015). doi:10.1016/j.bbamem.2015.02.014
28. Lam, R. S., Nahirney, D. & Duszyk, M. Cholesterol-dependent regulation of adenosine A(2A) receptor-mediated anion secretion in colon epithelial cells. *Exp. Cell Res.* **315,** 3028–35 (2009).
29. Charalambous, C. *et al.* Restricted collision coupling of the A2A receptor revisited: evidence for physical separation of two signaling cascades. *J. Biol. Chem.* **283,** 9276–88 (2008).
30. Bari, M., Paradisi, A., Pasquariello, N. & Maccarrone, M. Cholesterol-dependent modulation of type 1 cannabinoid receptors in nerve cells. *J. Neurosci. Res.* **81,** 275–283 (2005).
31. Bari, M., Battista, N., Fezza, F., Finazzi-Agrò, A. & Maccarrone, M. Lipid rafts control signaling of type-1 cannabinoid receptors in neuronal cells: Implications for anandamide-induced apoptosis. *J. Biol. Chem.* **280,** 12212–12220 (2005).
32. Bari, M. *et al.* Effect of lipid rafts on Cb2 receptor signaling and 2-arachidonoyl-glycerol metabolism in human immune cells. *J. Immunol.* **177,** 4971–80 (2006).
33. Nguyen, D. H. & Taub, D. Cholesterol is essential for macrophage inflammatory protein 1 beta binding and conformational integrity of CC chemokine receptor 5. *Blood* **99,** 4298–4306 (2002).
34. Nguyen, D. H. & Taub, D. D. Inhibition of chemokine receptor function by membrane cholesterol oxidation. *Exp. Cell Res.* **291,** 36–45 (2003).
35. Nguyen, D. H. & Taub, D. CXCR4 function requires membrane cholesterol: implications for HIV infection. *J. Immunol.* **168,** 4121–4126 (2002).
36. Gimpl, G., Burger, K. & Fahrenholz, F. Cholesterol as modulator of receptor function. *Biochemistry* **36,** 10959–74 (1997).
37. Harikumar, K. G. *et al.* Differential effects of modification of membrane cholesterol and sphingolipids on the conformation, function, and trafficking of the G protein-coupled cholecystokinin receptor. *J. Biol. Chem.* **280,** 2176–2185 (2005).
38. Potter, R. M., Harikumar, K. G., Wu, S. V. & Miller, L. J. Differential sensitivity of types 1 and 2 cholecystokinin receptors to membrane cholesterol. *J. Lipid Res.* **53,** 137–48 (2012).
39. Maguire, P. A. & Druse, M. J. The influence of cholesterol on synaptic fluidity, dopamine D1 binding and dopamine-stimulated adenylate cyclase. *Brain Res. Bull.* **23,** 69–74 (1989).
40. Pang, L., Graziano, M. & Wang, S. Membrane cholesterol modulates galanin-GalR2 interaction. *Biochemistry* **38,** 12003–12011 (1999).
41. Eroglu, C., Brugger, B., Wieland, F. & Sinning, I. Glutamate-binding affinity of Drosophila metabotropic glutamate receptor is modulated by association with lipid rafts. *Proc. Natl. Acad. Sci. U. S. A.* **100,** 10219–10224 (2003).
42. Kumari, R., Castillo, C. & Francesconi, A. Agonist-dependent signaling by group I metabotropic glutamate receptors is regulated by association with lipid domains. *J. Biol. Chem.* **288,** 32004–32019 (2013).
43. Colozo, A. T., Park, P. S. H., Sum, C. S., Pisterzi, L. F. & Wells, J. W. Cholesterol as a determinant of cooperativity in the M2 muscarinic cholinergic receptor. *Biochem. Pharmacol.* **74,** 236–255 (2007).
44. Michal, P., Rudajev, V., El-Fakahany, E. E. & Dolezal, V. Membrane cholesterol content influences binding properties of muscarinic M2 receptors and differentially impacts activation of second messenger

pathways. *Eur. J. Pharmacol.* **606,** 50–60 (2009).
45. Furukawa, H. & Haga, T. Expression of functional M2 muscarinic acetylcholine receptor in Escherichia coli. *J. Biochem.* **127,** 151–61 (2000).
46. Oates, J. *et al.* The role of cholesterol on the activity and stability of neurotensin receptor 1. *Biochim. Biophys. Acta* **1818,** 2228–33 (2012).
47. Monastyrskaya, K., Hostettler, A., Buergi, S. & Draeger, A. The NK1 receptor localizes to the plasma membrane microdomains, and its activation is dependent on lipid raft integrity. *J. Biol. Chem.* **280,** 7135–7146 (2005).
48. Levitt, E. S., Clark, M. J., Jenkins, P. M., Martens, J. R. & Traynor, J. R. Differential effect of membrane cholesterol removal on mu and delta opioid receptors: A parallel comparison of acute and chronic signaling to adenylyl cyclase. *J.Biol.Chem.* (2009).
49. Gaibelet, G. *et al.* Cholesterol content drives distinct pharmacological behaviours of micro-opioid receptor in different microdomains of the CHO plasma membrane. *Mol. Membr. Biol.* **25,** 423–35 (2008).
50. Lagane, B. *et al.* Role of sterols in modulating the human mu-opioid receptor function in Saccharomyces cerevisiae. *J. Biol. Chem.* **275,** 33197–200 (2000).
51. Huang, P. *et al.* Cholesterol reduction by methyl-β-cyclodextrin attenuates the delta opioid receptor-mediated signaling in neuronal cells but enhances it in non-neuronal cells. *Biochem. Pharmacol.* **73,** 534–549 (2007).
52. Xu, W. *et al.* Localization of the kappa Opioid Receptor in Lipid Rafts. *J. Pharmacol. Exp. Ther.* **317,** 1295–1306 (2006).
53. Gimpl, G., Reitz, J., Brauer, S. & Trossen, C. Oxytocin receptors: ligand binding, signalling and cholesterol dependence. *Prog. Brain Res.* **170,** 193–204 (2008).
54. Fahrenholz, F., Klein, U. & Gimpl, G. Conversion of the myometrial oxytocin receptor from low to high affinity state by cholesterol. *Adv Exp Med Biol* **395,** 311–319 (1995).
55. Klein, U., Gimpl, G. & Fahrenholz, F. Alteration of the myometrial plasma membrane cholesterol content with beta-cyclodextrin modulates the binding affinity of the oxytocin receptor. *Biochemistry* **34,** 13784–13793 (1995).
56. Rod, R., Segments, O. & Albert, D. Cholesterol Modulation of Photoreceptor Function in Bovine Retinal Rod Outer Segments. *J. Biol. Chem.* **265,** 20727–20730 (1990).
57. Mitchell, D. C., Straume, M., Miller, J. L. & Litman, B. J. Modulation of Metarhodopsin Formation by Cholesterol-Induced Ordering of Bilayer Lipids? *Biochemistry* **29,** 9143–9149 (1990).
58. Niu, S. L., Mitchell, D. C. & Litman, B. J. Manipulation of cholesterol levels in rod disk membranes by methyl-β-cyclodextrin: Effects on receptor activation. *J. Biol. Chem.* **277,** 20139–20145 (2002).
59. Pucadyil, T. J. & Chattopadhyay, A. Cholesterol modulates the antagonist-binding function of hippocampal serotonin1A receptors. *Biochim. Biophys. Acta* **1714,** 35–42 (2005).
60. Chattopadhyay, A., Jafurulla, M., Kalipatnapu, S., Pucadyil, T. J. & Harikumar, K. G. Role of cholesterol in ligand binding and G-protein coupling of serotonin1A receptors solubilized from bovine hippocampus. *Biochem. Biophys. Res. Commun.* **327,** 1036–41 (2005).
61. Pucadyil, T. J. & Chattopadhyay, A. Cholesterol modulates ligand binding and G-protein coupling to serotonin(1A) receptors from bovine hippocampus. *Biochim. Biophys. Acta* **1663,** 188–200 (2004).
62. Prasad, R., Paila, Y. D., Chattopadhyay, A. & Jafurulla, M. Membrane cholesterol depletion enhances ligand binding function of human serotonin1A receptors in neuronal cells. *Biochem. Biophys. Res. Commun.* **390,** 93–6 (2009).
63. Pucadyil, T. J. & Chattopadhyay, A. Cholesterol depletion induces dynamic confinement of the G-protein coupled serotonin(1A) receptor in the plasma membrane of living cells. *Biochim. Biophys. Acta* **1768,** 655–68 (2007).
64. Prasad, R., Paila, Y. D., Jafurulla, M. & Chattopadhyay, A. Membrane cholesterol depletion from live cells enhances the function of human serotonin(1A) receptors. *Biochem. Biophys. Res. Commun.* **389,** 333–7 (2009).
65. Sjögren, B., Hamblin, M. W. & Svenningsson, P. Cholesterol depletion reduces serotonin binding and

signaling via human 5-HT7(a) receptors. *Eur. J. Pharmacol.* **552,** 1–10 (2006).
66. Dahl, A. C. E., Chavent, M. & Sansom, M. S. P. Bendix: intuitive helix geometry analysis and abstraction. *Bioinformatics* **28,** 2193–4 (2012).
67. Guixà-González, R. *et al.* MEMBPLUGIN: studying membrane complexity in VMD. *Bioinformatics* **30,** 1478–1480 (2014).
